# A New Approach to Breast Specimen Orientation: Avoiding Pitfalls with the Specimen Plate Concept

**DOI:** 10.3390/curroncol31080342

**Published:** 2024-08-10

**Authors:** András Drozgyik, Tamás Szabó, György Kovács, Dániel Kollár, Tamás F. Molnár

**Affiliations:** 1 Department of Burns and Plastic Surgery, Petz Aladár University Teaching Hospital, 9024 Győr, Hungary; 2 Doctoral School of Clinical Sciences, University of Pécs Medical School, 7624 Pécs, Hungary; tfmolnar@gmail.com; 3 Department of Pathology, Petz Aladár University Teaching Hospital, 9024 Győr, Hungary; 4 Multidisciplinary Doctoral School of Engineering Sciences, Széchenyi István University, 9026 Győr, Hungary; 5 Kirurgkliniken, Värnamo Sjukhus, 331 56 Värnamo, Sweden; 6 Department of Operational Medicine, University of Pécs Medical School, 7624 Pécs, Hungary

**Keywords:** breast cancer, resection margins, specimen orientation

## Abstract

Accurate specimen marking is crucial during breast cancer surgery to avoid misorientation, which can lead to inadequate re-excision and tumor recurrence. We studied the marking methods at various breast cancer centers to create a tool that would prevent specimen misorientation. An online questionnaire was used to survey marking procedures at major breast cancer centers in Hungary, and a tool was developed using a troubleshooting method. Twelve out of twenty units responded (60%). Nine use an institutionally standardized marking system. Less than half of the surgical teams found specimen mammograms to be unambiguous. In more than 70% of departments, pathologists were uncertain about breast specimen orientation. Ambiguous marking methods caused orientation errors in half of the cases, while unclear marking directions caused the rest. Most pathologists (85%) and surgeons (75%) believed that coronal plane specimen mammography would help solve the problem. A plastic specimen plate has been developed to anchor breast tissue to a coronal breast scheme as seen in mammography images, providing clear localization information throughout the surgical process. There is a lack of standardization in breast specimen orientation and marking in Hungary. An optimized orientation toolkit is being developed to ensure consistent interpretation of specimen mammograms by surgeons and pathologists.

## 1. Introduction

The aim of breast-conserving surgery is complete tumor removal with satisfactory cosmetic results and adequate oncologic radicality, characterized by safe surgical margins [1].

Advances in screening and neoadjuvant therapies have increased the detection of non-palpable breast tumors, requiring precise preoperative lesion marking for accurate resection and reliable specimen orientation for intraoperative radiologic evaluation of specimens. Clear communication and coordination among surgeons, pathologists, and radiologists is critical, as is accurate marking of resection margins to ensure precise orientation of excised breast tissue during pathological examination [2,3]. Misorientation of specimens can lead to errors in margin assessment, potentially compromising oncologic outcomes and increasing the risk of local recurrence [4].

Current surgical guidelines emphasize the importance of standardized protocols for specimen orientation, requiring detailed information about the anatomical extent of the primary surgery and the location of the tumor within the excised specimen [1,5]. Despite these guidelines, variability in specimen marking and orientation practices remains a significant challenge in breast cancer surgery.

In response to these challenges, our study sought to evaluate the current techniques used for breast specimen orientation across Hungarian breast cancer treatment centers. Our goal was to assess the radiologists’, pathologists’, and surgeons’ satisfaction with existing practices and to identify areas for improvement. Based on the findings from this survey, we have developed a novel tool to improve the accuracy of specimen orientation and the overall quality of surgical and pathological processes in breast-conserving surgery.

## 2. Materials and Methods

A review was performed to assess the techniques used for breast specimen orientation from the operating field to microscopic sectioning in Hungarian breast cancer treatment departments.

The review process involved a comprehensive analysis of current practices across multiple institutions, focusing on the methodologies used for specimen marking and orientation. An online questionnaire was developed to collect data from surgeons, radiologists, and pathologists regarding their breast cancer specimen orientation protocols. The questionnaire was designed to gather detailed information about the specific marking methods used, their perceived effectiveness, and any challenges encountered. The review also assessed radiologists’, pathologists’, and surgeons’ satisfaction with these techniques (Table 1).

To ensure comprehensive coverage and representation of practices across Hungary, surgeons, radiologists, and pathologists from the top 20 breast cancer centers were surveyed using the online questionnaire above (Regional Science and Research Ethics Committee permission: 76-1-19/2021). These centers were selected based on their volume of breast cancer surgeries and their contributions to breast cancer care in Hungary (population: 9.7 million, with an estimated breast cancer incidence of 8233 in 2019 [6]). Twelve out of twenty departments responded as shown below. Responses were collected and analyzed to identify common practices and areas where discrepancies or challenges were reported. All centers agreed to have their reported practices evaluated anonymously. Table 2 contains the responding centers. From each center, a breast surgeon, a mammologist-radiologist, and a pathologist responsible for breast cancer diagnosis completed the questionnaire, representing their departmental practices:

## 3. Results

Marking with orientation sutures indicating the four anatomical directions (caudal-cranial, lateral, medial; length of strand signaling each of the courses) was the standard procedure used in all responding surgical departments. Three quarters of the responding centers (9 out of 12) use a uniform system for orienting and marking breast tissue. In a quarter of the departments, an orienting schematic drawing accompanies the specimen from the operating room to the path lab. 

Specimen mammography images were unambiguous for less than half of the responding surgeons, while obvious for all radiologists. None of the reporting centers take coronal plane specimen mammography images according to the radiologists’ response. More than 70% of responding pathology departments experience difficulty or ambiguity in the orientation of breast tissue specimens in around 5–10% of the cases. From the pathologists’ point of view, in half of the cases, the reason for orientation errors is that the way of marking the excisions is ambiguous. Suture length is a common source of misunderstanding. In the remaining ambiguous cases, the directions of the markings are not clear. Many reported failures due to loss of suture during the specimen mammography and transport to pathology. Ten out of the twelve responding pathology departments and 75% of responding surgical teams believe that coronal plane specimen mammography would be useful, especially in cases of non-palpable tumor resections. In two reporting centers, pathologists do not have access to specimen mammography images.

Two thirds of the surgeons believe that an intraoperative photograph of the excision cavity and specimen would be helpful for orienting the re-resection. The most common sources of inadequate markings or even errors in existing orientation techniques, such as suture or clip markings, were reported in the notes section of the questionnaire. Three-way suture or clip markers become dislodged due to the irregular shape and consistency of the resected breast tissue. This may result in displacement or even loss of the marker. The orientation of the mammography image is often unclear to the surgeon, leading to incorrect site identification (Table 3).

As a result of the survey, the basic requirements for an unbiased presentation platform were identified. It must securely fix the specimen and reproduce the original, inpatient position of the removed tissue mass with a high degree of reproducibility. The platform must be radio-translucent, as specimen mammography is an integral part of the protocol. Ideally, it is sterile to facilitate intraoperative maneuvers and the work of the scrub nurse.

**Figure 1:**
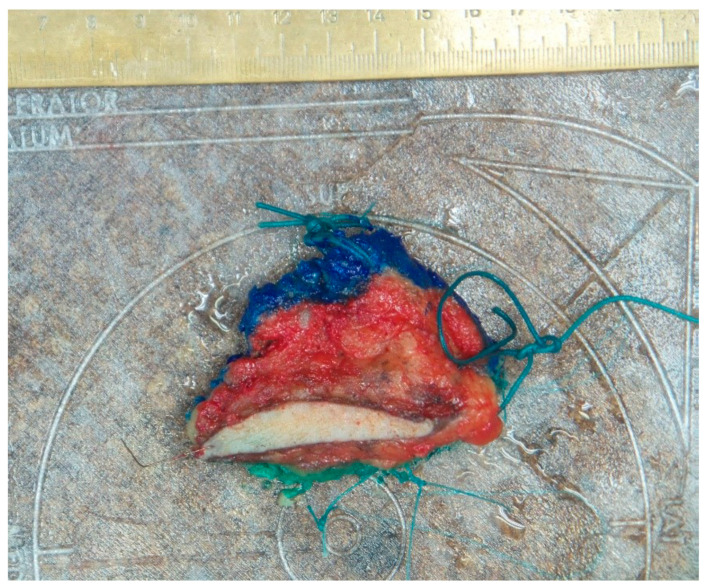
Oriented excised breast tissue specimen in anatomically correct position on the specimen plate. Ambiguity of the pathological color marking of the surfaces demonstrated.

**Figure 2:**
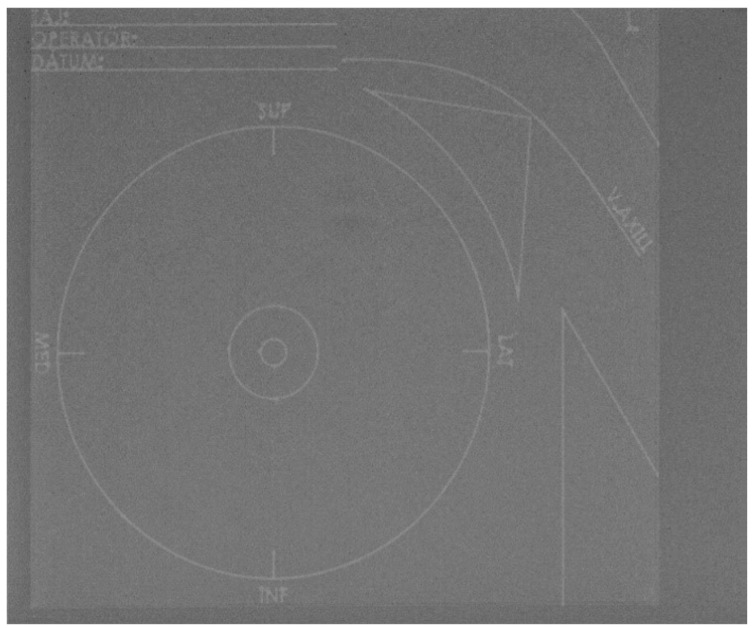
Mammographic image of the specimen plate. Anatomy and location are clear without a disturbing X-ray positive shadow.

**Figure 3:**
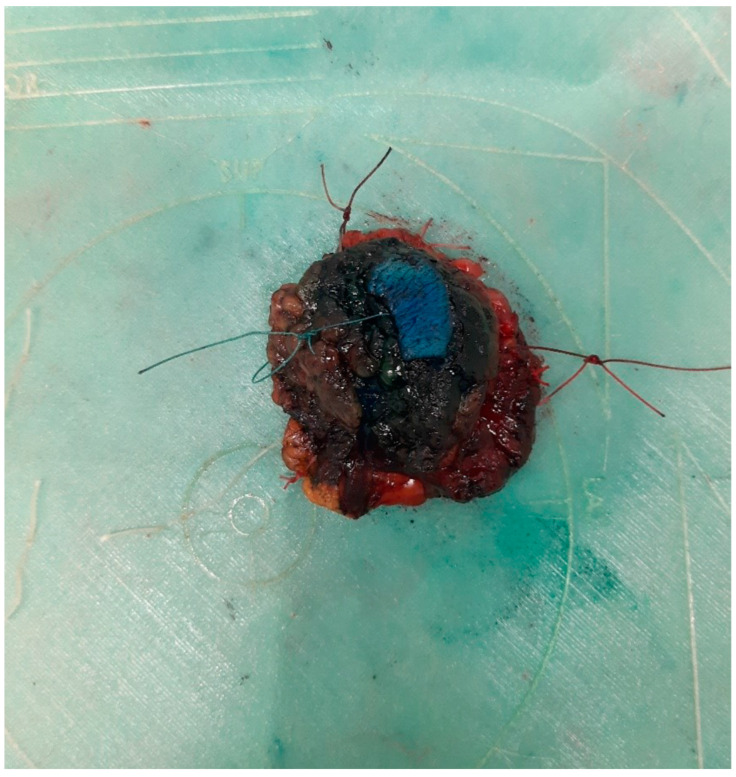
Correctly oriented excised breast tissue specimen on the specimen plate (radio-guided occult lesion localization surgery).

**Figure 4:**
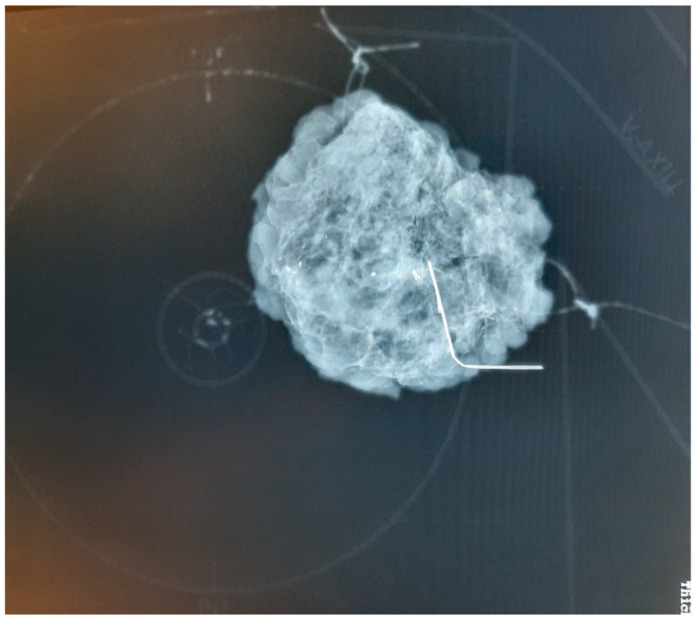
Mammographic image of the oriented breast tissue specimen with guide-wire tumor localization on the specimen plate. The additional information of the plate is the high-fidelity localization of the specimen within the breast. Traditional suture marking (long = lateral; short = superior) is also used, with a thick yarn that creates an X-ray shadow.

In order to meet the above detailed requirements, a 3D printed (For further details, see Appendix A) plastic specimen plate was built (Figure 1, Figure 2, Figure 3 and Figure 4), which allows the oriented anchoring of the breast tissue specimen representing the original in vivo situation. In this way, the removed specimen is completely consistent with the original layout in the patient. The plate is designed to mimic the female breast and axillary scheme so that the location and direction of the specimen can be unambiguously determined.

This scheme is clearly visible on mammographic images, but the plate does not have a disturbing X-ray-positive shadow (Figure 2 and Figure 4). This plate provides mammographic images of breast specimens in the coronal plane, which are easier to understand and more reliable for surgeons and pathologists.

## 4. Discussion

**Figure 5:**
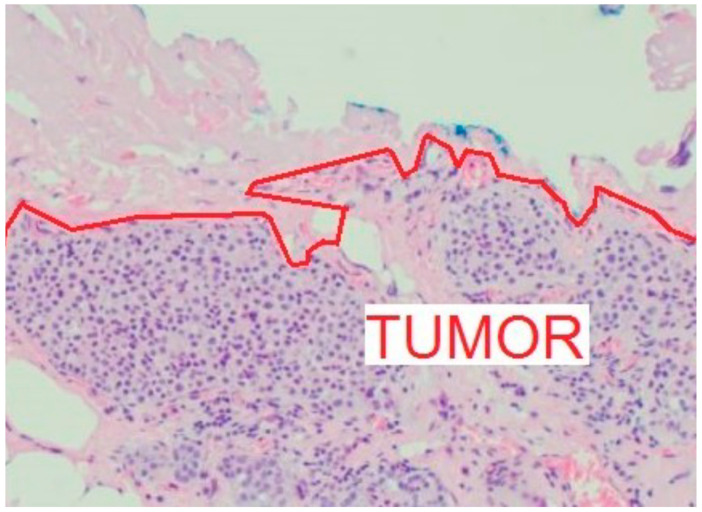
Due to the irregular surface of both the excised specimen (marked with blue ink) and the tumor (marked with a red line), the distance between them varies along the examined margins. Correct measurement of margin width is varying and ambiguous (photomicrograph courtesy of Tamás Szabó, MD, USA).

Current evidence regarding the re-excision rate following breast-conserving surgery is uncertain, but the literature suggests that a significant proportion of cases, ranging from 4% to 30%, require further intervention. However, the incidence of re-excision has decreased with the increasing use of oncoplastic techniques [7,8]. Notably, approximately half of these re-operations are performed in patients with negative surgical margins [9,10,11]. A negative surgical margin is defined as no tumor on ink, where ink is used to mark the sides of the histological specimen (Figure 5).

Pathologically negative surgical resection margins depend on several factors, including the number of sections examined and the direction and technique of pathological sectioning. The type of breast cancer, its stromal invasion (for instance the lack of E-cadherin), or its intraductal spread can significantly affect the assessment of resection margins due to the irregularities of the tumor edge [12]. Other important factors include type of transport and storage until pathological processing, orientation techniques, and mammography with specimen compression. In a compressed breast tissue specimen, the specimen dimensions are reduced in the compression direction to approximately half of the normal width and doubled in the perpendicular direction. It is estimated that a complete examination of the edge surfaces of an average spherical lumpectomy specimen would require 3000 sections [13].

The complexity of the procedure explains why nearly half of all re-excisions result in negative surgical margins in the final report. Despite consensus recommendations, the current practice is to consider a surgical margin of approximately 1 mm or less as an indication for re-excision to achieve R0 status. It is expected that a larger negative margin will improve disease outcomes [14,15]. Naturally, local recurrence and prognosis are influenced not only by surgical margins but also by factors such as primary tumor size, grade, lymph node metastases, stage, proliferative activity, and estrogen receptor expression [16].

Increased screening and effective neoadjuvant treatments have resulted in a higher incidence of non-palpable breast tumors [17,18], making preoperative marking critical for accurate resection. Wire-guided localization, the initial method for marking non-palpable tumors, continues to be utilized despite its limitations. However, radio-guided localization is also gaining widespread adoption, and new techniques employing magnetic, radar, ultrasound or radiofrequency technologies are also emerging [19,20,21]. The rising incidence of non-palpable tumors also underscores the importance of specimen orientation due to the necessity of intraoperative radiological examination for evaluating the radiological centrality of the tumor within the specimen.

Current guidelines require a protocol for breast tissue specimen marking to ensure clear orientation of the excised specimen during breast cancer surgery [1,5]. Available methods for breast specimen orientation include suture marking, clip marking, ink marking, and schematic drawing [22]. In cases of inadequate or positive surgical margins, re-excision in the direction of the affected margin is required. This results in better cosmetic outcomes by minimizing the removal of intact tissue compared to complete wound cavity excision [23], allowing for larger volume removal in the necessary direction [24]. This requires accurate information about the anatomical extent and dimensions of the primary surgery and the location of the tumor within the specimen. Therefore, specimen orientation procedures are of paramount importance.

The survey revealed a general dissatisfaction with the status quo in the OR–radiology–pathology chain. Notable discrepancies were observed in the responses from pathologists, radiologists, and surgeons regarding specimen orientation and marking methods. Even within the same institutions, there seems to be a lack of consensus on the adequacy and interpretation of these methods. This divergence points to potential misunderstandings and varying approaches to similar challenges, emphasizing the need for standardized protocols and enhanced communication among multidisciplinary teams to ensure more consistent and accurate specimen handling and assessment.

There are identifiable general trends. There is a common trend that paves the way for the solution. The keywords are recognizable topographical points that align with the actual anatomy of the individual patient and non-radio opacity of the platform. Multi-directional intraoperative mammography, supplemented by an antero-posterior image in the coronal plane, provides an optimal foundation for both pathological and surgical orientation. The task is to present the excised tissue mass to the pathologist in an unambiguous manner.

Since excisions must usually be made from the chest wall to the pectoral fascia and up to the subcutaneous tissue, it is important to mark and locate the tumor in the coronal plane. Demonstration plates intend to reduce information loss and increase patient/procedural safety in the surgeon–pathologist–oncologist chain [2]. However, this type of specimen presentation has not been available in breast oncology up to now. The presented concept and method for the breast specimen plate (patent pending), developed in accordance with the clinical needs of the daily practice explored in our survey, appear to offer a viable solution to the problem described.

In acknowledging the limitations of this study, several factors must be considered. Firstly, the use of the specimen plate, while innovative, is somewhat time-consuming. Although it offers notable advantages, such as improved orientation accuracy and clearer mammographic images, the plate also adds several minutes to the procedure. A comparative study is currently underway at our institution to assess the efficacy of suture marking versus the specimen plate. However, a multicentric approach would further enhance the generalizability and reliability of the findings. Therefore, despite the significant advancements represented by the specimen plate, additional research involving multiple centers is crucial to validate its broader applicability and its impact on surgical and pathological outcomes.

## 5. Conclusions

A need to improve quality control in an important subset of breast cancer surgery has been identified. The orientation of the specimen mammography image in the coronal plane, supplemented by the breast scheme of the plate, may offer clearer understanding for pathologists and serve as an orientation tool for the breast surgeon during re-excision or re-operation. The specimen plate developed aims to improve the accuracy and synchronization of mammography–surgery–pathology processing. It effectively addresses the limitations of traditional orientation methods, thereby improving the overall accuracy and reliability of breast cancer specimen analysis.

## Figures and Tables

**Table 1:** Online questionnaire for surgeons, radiologists, and pathologists about their breast cancer specimen orientation protocols in Hungary.

What type of marking is used at your institution to orient specimens for breast-conserving surgery? Multiple answers are possible. Possible answers: Suture markings/Clip markings/Orientation scheme or drawing/Other method In cases of non-palpable breast tumors, is the orientation of the specimen always unambiguous to you based on the intraoperative specimen mammography image? Possible answers: Yes/No In cases where specimen mammography was ambiguous, what percentage showed ambiguity? (Question for surgeons)/How often have you encountered ambiguity in specimen orientation (Question for radiologists and pathologists)? Possible answers: <10%/10–50%/> 50% What is the main cause of uncertainty in specimen orientation? Possible answers: Unclear Directions of Markings/Ambiguous Marking Methods/Other In which directions or planes is the intraoperative specimen mammography taken? Possible answers: Bi-directional imaging is taken (cranio-caudal and medio-lateral)/Antero—posterior direction (coronal or frontal plane) is also taken If only bi-directional (cranio-caudal and latero-medial images are taken), would you find an image in antero-posterior direction (coronal or frontal plane) useful to accurately determine the direction of re-excision if needed? Possible answers: Yes/No In cases of non-palpable tumors, where preoperative localization is required, what is more important in determining the direction of a possible re-excision? (Question for surgeons) Possible answers: Mammographic image for the surgeon/Consultation with the radiologist If re-excision is required due to inadequate or compromised surgical margins/for pathological assessment, would you consider an intraoperative image of the excision cavity and specimen to be helpful in the orientation of the re-resection? Possible answers: Yes/No Is the orientation protocol uniform at your institution? Possible answers: Yes/No If orientation at your institution is not uniform, would you consider a uniform orientation method useful? Possible answers: Yes/No Would you find it useful to have a nationally uniform marking and orientation method? Possible answers: Yes/No Do you use specimen mammography for pathological assessment in cases of non-palpable breast tumors? (Question for pathologists) Possible answers: Yes/No Describe the details of the marking system used at your institution. (For example, in the case of suture marking: double, short suture = superior; single, long suture = lateral)

**Table 2:** List of major breast cancer centers that participated in the survey.

Department of Surgery, Petz Aladár University Teaching Hospital, Győr, Hungary; Department of Breast and Sarcoma Surgery, National Institute of Oncology, Budapest, Hungary; Department of Surgery, Transplantation and Gastroenterology, Semmelweis University, Faculty of Medicine, Budapest, Hungary; Department of Surgery, University of Pécs, Budapest, Hungary; Department of Surgery, Uzsoki Street Hospital, Budapest, Hungary; Department of Surgery, Hungarian Defense Forces Medical Centre, Budapest, Hungary; Department of Plastic Surgery, South Buda Central Hospital Szent Imre University Teaching Hospital, Budapest, Hungary; Department of Surgery, University of Szeged, Szeged, Hungary; Department of Surgery, University of Debrecen, Debrecen, Hungary; Department of Obstetrics and Gynecology, Central Hospital of Southern Pest National Institute of Hematology and Infectious Diseases, Szent István Hospital, Budapest, Hungary; Department of Surgery, Bajcsy-Zsilinszky Hospital and Clinic, Budapest, Hungary; Surgical Center, Ferenc Csolnoky County Hospital Veszprém, Hungary;

**Table 3:** Summary of questionnaire results by responder group.

Questions ^1^	Answers	Responder Groups		
		Pathologists	Surgeons	Radiologists
1. Marking methods used for specimen orientation	Suture markings ^2^	100%	100%	100%
	Clip markings	11.1%	8.3%	0%
	Orientation drawings/schematics	22.2%	25%	16.7%
	Other Methods (including various types of thread markings and schematic drawings)	11.1%	8.3%	0%
2. Use of specimen mammograms for pathological assessment in case of non-palpable tumors	Yes No	77.8% 22.2%	n/a	n/a
3. Specimen mammography clarity	Unambiguous	25%	41.7%	100%
	Ambiguous	75%	58.3%	0%
4. Percentage of ambiguity in specimen mammograms where it exists (for surgeons)/percentage of ambiguity in specimen orientation (for radiologists and pathologists)	<10%	71.4%	50%	66.7%
	10–50%	28.6%	50%	33.3%
	>50%	0%	0%	0%
5. Common causes of misunderstanding (for radiologists and pathologists)	Unclear Directions of Markings	50%	n/a	80%
	Ambiguous marking methods	50%		20%
6. Imaging directions	Two-Directional (cranio-caudal and medio-lateral)	100%	100%	100%
	Antero-Posterior (A-P) Direction (coronal plane)	0%	0%	0%
7. Additional coronal plane imaging utility	Beneficial	83.3%	75%	n/a
	Not beneficial	16.6%	25%	
8. Significant factor in determining the direction of a re-excision for non-palpable tumors	Mammographic image for the surgeon	n/a	25%	n/a
	Consultation with the radiologist		75%	
9. Intraoperative photographs	Beneficial	66.7%	66.7%	n/a
	Not beneficial	33.3%	33.3%	
10. Standardization of protocol exists	Yes	75%	75%	75%
	No	25%	25%	25%
11. Usefulness of standardized protocol (in the absence of standardization)	Found useful	87.5%	66.7%	n/a
	Not found useful	12.5%	33.3%	
12. Nationwide standardization	Found useful	87.5%	66.7%	n/a
	Not found useful	12.5%	33.3%	
13. Types of suture markings used (open-ended answer)	The suture marking method for orientation of breast-conserving surgical specimens primarily involved the use of sutures of varying lengths and placement to indicate different anatomical directions. The commonly reported marking scheme included Short (superior): Two short sutures indicating the superior (upper) margin of the specimen. Long (lateral): One long suture marking the lateral (outer) margin. Medium (medial): One short suture denoting the medial (inner) margin. Different colors for Mammillary-Central, as indicated by various methods depending on the institution, often involving different colored sutures. It was noted that some institutions employed variations in their marking techniques. These variations included the use of either multiple or single sutures to differentiate the oriented sides of the specimen.			

^1^ Questionnaires were customized to suit the specific respondent groups. ^2^ See below in Question 13.

## Data Availability

Data are contained within the article.

## Appendix A. 3D Printing Details

SPECIMEN PLATE—TECHNICAL SPECIFICATIONS OF PRODUCTION

Specimen plate dimensions: 209 mm × 199.5 mm × 1.2 mm

1. The plate is made using a Creality Ender 5 3D printer (Creality, Shenzhen, China)
2. A layer thickness of 0.1 mm was used to create a solid yet braided structure. This allows the material to be shaped like a textile but be more flexible.
3. The material is TPU A95 synthetic resin. This material holds the stitches used to attach the specimen securely, maintaining proper tensile strength even if only a small bit of the plate is occupied by the stitch.
4. Printing parameters:
  - Print speed: 30 mm/s
  - Printing temperature: 240 °C
  - Nozzle diameter: 0.6 mm
